# Pseudo Chediak-Higashi anomaly in a diffuse large B-cell lymphoma with hemophagocytic lymphohistiocytosis

**DOI:** 10.1007/s44313-024-00052-4

**Published:** 2025-01-08

**Authors:** Biyun Yi, Mengying Zeng

**Affiliations:** Department of Clinical Laboratory, Changde Hospital, Xiangya School of Medicine, Central South University (The First People’s Hospital of Changde City), No. 388, People’s East Road, Changde, Hunan Province 415000 China

**Keywords:** Pseudo Chediak-Higashi, Diffuse large B cell lymphoma, Hemophagocytic lymphohistiocytosis

A 75-year-old male was admitted to the hospital with a recurrent fever lasting over a month. Physical examination revealed involuntary tremors in both hands and multiple rashes with apparent bleeding in both lower limbs. Laboratory test results showed leukocytes at 13.1 × 10^9^/L, hemoglobin at 114 g/L, platelets at 108 × 10^9^/L, and elevated levels of lactate dehydrogenase, triglycerides(3.66 mmol/L), ferritin (2745 ng/ml), and soluble interleukin-2 receptor (sIL-2R; 34,170 pg/ml). Fluorodeoxyglucose positron emission tomography (FDG-PET) revealed splenomegaly with increased tracer uptake. Bone marrow aspiration showed occasional hemophagocytosis, and approximately 13% of total lymphocytes were abnormal, containing vacuolated pink inclusions of varying sizes, referred to as pseudo-Chediak-Higashi anomalies (Fig. 1A-D). These cytoplasmic inclusions were negative for myeloperoxidase staining (Fig. 1E) and positive for periodic acid-Schiff staining (Fig. 1F). Flow cytometric analysis revealed that approximately 7.45% of the abnormal lymphocytes were positive for CD19, CD20, CD22, and CD79b, exhibited restricted expression of immunoglobulin kappa light chain, and were negative for CD5 and CD10. Cytogenetic studies showed a complex karyotype of 45,X,-Y [4] / 45, idem, add(1)(q32), dic(2;9)(p13;p22), t(2;13)(q11.2;p11.2), del(6)(q21), -7, -8, add(9)(p13), del(16)(q22), der(17)t(1;17)(q12;p13), + mar1, + mar2, + mar3 [8] / 46,XY [8]. A bone marrow biopsy confirmed diffuse large B-cell lymphoma (DLBCL). Interestingly, a mucosal biopsy of the gastric body also suggested DLBCL. Consequently, a diagnosis of diffuse large B-cell lymphoma with hemophagocytic lymphohistiocytosis (HLH) was established, and the patient was treated with induction chemotherapy consisting of cytarabine and idarubicin. Twenty-three days later, the patient developed severe pneumonia, and treatment was discontinued.

**Fig.1:**
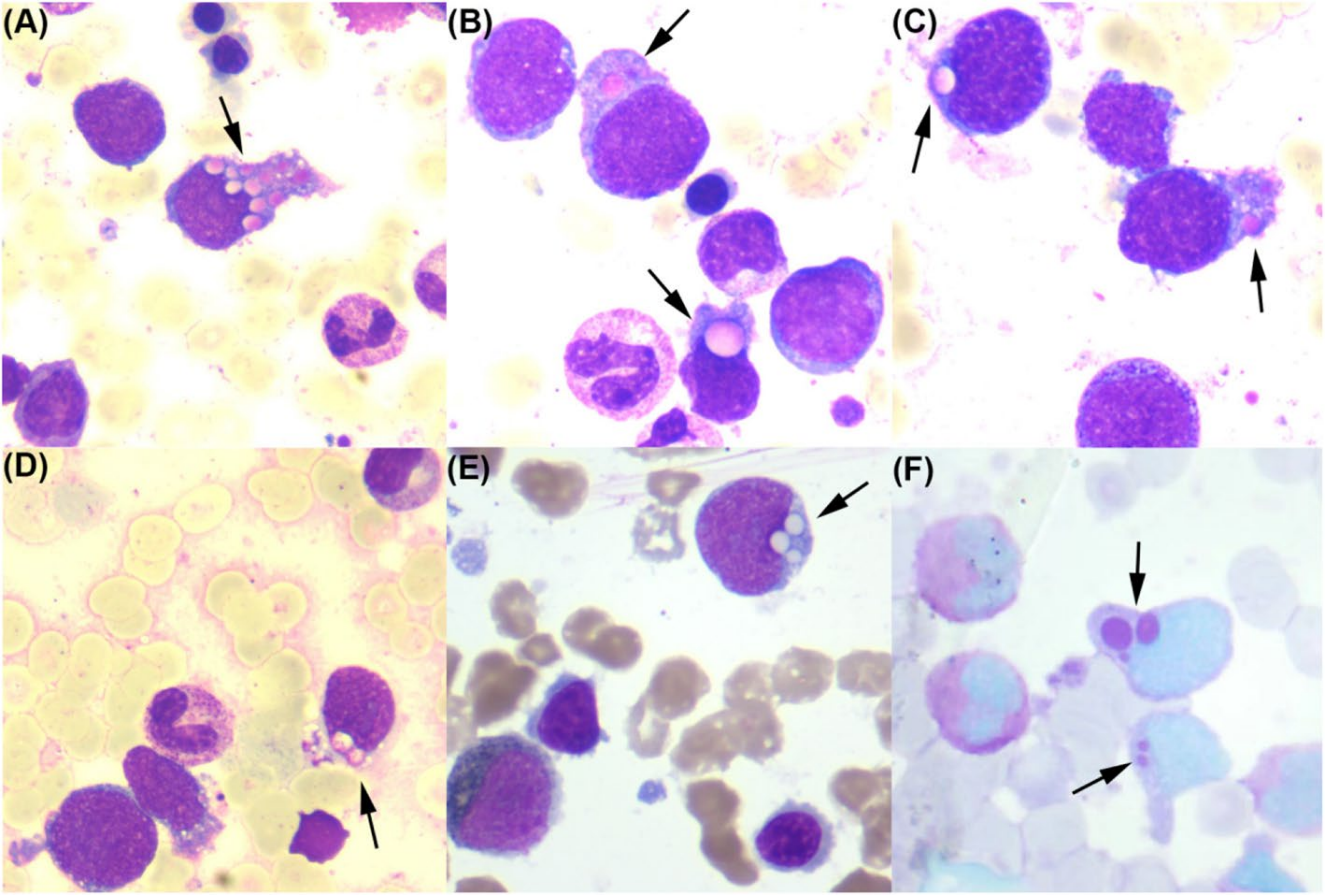
**A**-**D** A few vacuolated pink inclusions of different sizes were found in the cytoplasm of lymphoma cells in bone marrow aspirate (black arrow, Wright–Giemsa staining; × 1000).These inclusions, known as pseudo-Chédiak-Higashi anomalies, are rare in mature B-cell tumors and draw attention to the unique morphological characteristics of these cells. **E** These inclusions were negative for myeloperoxidase staining (black arrow, POX staining; × 1000). **F** These inclusions were positive for periodic acid-Schiff staining (black arrow, PAS staining; × 1000)

Pseudo-Chédiak-Higashi inclusions have been reported in acute myeloid leukemia, acute lymphoblastic leukemia, and chronic myeloid leukemia, appearing in blasts and mature neutrophils [1–4]. To the best of our knowledge, these have only been described in two cases with mature B-cell tumors. Stoieva et al. reported a case of marginal zone lymphoma with pseudo-Chédiak-Higashi inclusions [5], and Yan et al. described granular inclusions in DLBCL [6]. Notably, in the present case, we observed vacuolated rather than granular pink inclusions within DLBCL lymphocytes, marking the first reported instance of such findings. Although pseudo-Chédiak-Higashi inclusions are relatively easy to identify in bone marrow morphology, they should not be considered evidence of myeloid lineage to guide initial treatment. Additionally, in this case, DLBCL was accompanied by hemophagocytic lymphohistiocytosis (HLH), coexisting with pseudo-Chédiak-Higashi inclusions and a complex karyotype, increasing the complexity of diagnosis and treatment. Timely identification, precise diagnosis, and prompt treatment are essential to improve patient outcomes. However, the mechanisms underlying formation of these vacuolated pink inclusions require further investigation.

## Data Availability

No datasets were generated or analysed during the current study.
